# Impact of Obesity on Short-Term Outcomes in Patients Undergoing Retroperitoneal Laparoscopic/Retroperitoneoscopic Adrenalectomy for Benign or Malignant Adrenal Diseases: A Meta-Analysis

**DOI:** 10.3390/medicina61010106

**Published:** 2025-01-13

**Authors:** Maurizio Zizzo, Andrea Morini, Magda Zanelli, Chiara Grasselli, Francesca Sanguedolce, Sze Ling Wong, Munyaradzi G. Nyandoro, Andrea Palicelli, Giuseppe Broggi, Nektarios I. Koufopoulos, Lucia Mangone, Angelo Cormio, Rosario Caltabiano, Antonino Neri, Massimiliano Fabozzi

**Affiliations:** 1 Surgical Oncology Unit, Azienda Unità Sanitaria Locale-IRCCS di Reggio Emilia, 42123 Reggio Emilia, Italy; andrea.morini@ausl.re.it (A.M.); massimiliano.fabozzi@ausl.re.it (M.F.); 2 Pathology Unit, Azienda Unità Sanitaria Locale-IRCCS di Reggio Emilia, 42123 Reggio Emilia, Italy; magda.zanelli@ausl.re.it (M.Z.); andrea.palicelli@ausl.re.it (A.P.); 3 Cardiovascular Medicine Unit and Secondary Hypertension Center, Azienda Unità Sanitaria Locale-IRCCS di Reggio Emilia, 42123 Reggio Emilia, Italy; chiara.grasselli@ausl.re.it; 4 Pathology Unit, Azienda Ospedaliero-Universitaria, Ospedali Riuniti di Foggia, 71122 Foggia, Italy; francesca.sanguedolce@unifg.it; 5 General and Endocrine Surgery, Royal Perth Hospital, Perth, WA 6000, Australia; szeling.wong@health.wa.gov.au (S.L.W.); munyaradzi.nyandoro@health.wa.gov.au (M.G.N.); 6 General and Endocrine Surgery, St. John of God Murdoch Hospital, Murdoch, WA 6150, Australia; 7 General and Endocrine Surgery, Fiona Stanley Hospital, Murdoch, WA 6150, Australia; 8 Department of Medical and Surgical Sciences and Advanced Technologies “G.F. Ingrassia”, Anatomic Pathology, University of Catania, 95123 Catania, Italy; giuseppe.broggi@phd.unict.it (G.B.); rosario.caltabiano@unict.it (R.C.); 9 Second Department of Pathology, Medical School, National and Kapodistrian University of Athens, Attikon University Hospital, 15772 Athens, Greece; nkoufo@med.uoa.gr; 10 Epidemiology Unit, Azienda Unità Sanitaria Locale-IRCCS di Reggio Emilia, 42123 Reggio Emilia, Italy; lucia.mangone@ausl.re.it; 11 Urology Unit, Azienda Ospedaliero-Universitaria Ospedali Riuniti di Ancona, Università Politecnica Delle Marche, 60126 Ancona, Italy; a.cormio@pm.univpm.it; 12 Scientific Directorate, Azienda Unità Sanitaria Locale-IRCCS di Reggio Emilia, 42123 Reggio Emilia, Italy; antonino.neri@ausl.re.it

**Keywords:** adrenal gland, adrenalectomy, laparoscopy, surgery, obesity, body mass index, outcomes

## Abstract

*Background and Objectives*: Retroperitoneal laparoscopic adrenalectomy (RLA) is one of two laparoscopic procedures used to treat benign and malignant adrenal diseases. Obesity in patients undergoing minimally invasive adrenal surgery is a frequently discussed topic. Our meta-analysis aimed to provide updated evidence by comparing intraoperative and perioperative outcomes on non-obese (NOb) and obese (Ob) patients who underwent RLA due to benign or malignant disease. *Materials and Methods*: We performed a systematic review following the Preferred Reporting Items for Systematic Reviews and Meta-Analyses (PRISMA) guidelines. PubMed/MEDLINE, Scopus, Web of Science (Science and Social Science Citation Index), and Cochrane Library (Cochrane Database of Systematic Reviews, Cochrane Central Register of Controlled Trials (CENTRAL)) databases were used to identify articles of interest. The meta-analysis was performed using RevMan [Computer program] Version 5.4. *Results*: The four included comparative studies (809 patients: 552 NOb versus 257 Ob) covered an approximately 15-year-study period (2007–2022). All the included studies were observational in nature. By comparing the Ob and NOb groups, shorter operative time and lower overall postoperative complication rates in the NOb population were recorded through the meta-analysis. Considering the subgroup analysis (BMI ≥ 30 kg/m^2^), just the operative time maintained statistical significance. *Conclusions*: Obesity did not appear to impact RLA safety and effectiveness. Due to important biases (small overall sample size and few analyzed events), the interpretation of our results must be a careful one. Later randomized and multi-center trials may help the confirmation of our results.

## 1. Introduction

The European Society of Endocrinology, the European Society of Endocrine Surgeons, and the American Association of Endocrine Surgeons agreed on minimally invasive surgery being the gold standard in many surgical adrenal disorders [1,2,3,4,5,6]. The 1992 study by Gagner et al. analyzed the first cases of laparoscopic adrenalectomy and the related results [7]. Later, many other studies highlighted the significant advantages of minimally invasive surgery, compared to the conventional approach: lower rates of postoperative morbidity and mortality, less frequent overall and major complications, reduced postoperative pain, shorter hospital stay, and better scars due to surgery [1,2,3].

Laparoscopy may have either a transperitoneal or retroperitoneal nature [1,2,3]. At present, transperitoneal laparoscopic adrenalectomy (TLA) is the chosen method [1,2,3]. TLA makes the overall view of the adrenal lodge and the surrounding area easier and allows enough working space in larger lesions [1,2,3,8]. The examination of the abdominal cavity leads to a preference for the transabdominal method, which facilitates the concurrent surgical management of other associated abdominal conditions in the course of intervention [1,2,3,8]. Moreover, when difficult dissection or intraoperative hemorrhage occurs, this approach allows for a timely shift to open surgery [1,2,3,8].

Retroperitoneal laparoscopic (retroperitoneoscopic) adrenalectomy (RLA) was first described by Mercan et al. in 1995, although it was promoted and popularized by Walz et al. in 2006 [9,10,11]. Its advantages over TLA include overlooking the intraabdominal cavity, immediately approaching the adrenal gland, no manipulation of intraabdominal viscera, and no repositioning of patients in case of bilateral procedures [1,2,3,8]. Different studies have underlined that RLA leads to fewer complications, shorter hospitalization, reduced hemorrhage, and shorter surgical times [1,2,3]. However, from a technical perspective, RLA also is more challenging due to the smaller working space [1,2,3,8]. Among the risk factors related to RLA, some authors stress the role of the patient’s obesity [1,2,3,8].

At present, experts often discuss the impact of obesity after general abdominal [12,13,14,15,16,17,18] and adrenal surgery [19,20,21,22]. Some studies have shown how obesity may play a detrimental role in patient outcomes of abdominal surgery [12,15,19,21]. As a matter of fact, postoperative morbidity rates in obese patients are higher compared to non-obese ones [12,15,19,21]. On the other hand, some studies identified a poor association between obesity and the onset of postoperative complications, underlining a preservative impact of obesity on postoperative mortality after surgery, the so-called “obesity paradox” [13,16,17,18,22]. Unfortunately, most studies have focused on the surgery of intraperitoneal organs or peculiar types of surgery (i.e., bariatric surgery), ruling out surgical procedures on retroperitoneal structures such as adrenal glands [20].

Our meta-analysis aimed to provide updated evidence through a comparison between intraoperative and perioperative outcomes among non-obese (NOb) and obese (Ob) patients who underwent RLA for benign or malignant adrenal diseases.

## 2. Materials and Methods

As our meta-analysis was based on previously published studies, and no original patient population data were added, approval by Ethics committee and informed patient consent were not required.

The present meta-analysis was performed following the Preferred Reporting Items for Systematic Reviews and Meta-Analyses (PRISMA) statement and guidelines [23]. Furthermore, our systematic review was not registered in a public registry.

### 2.1. Search Strategy

Articles of interest were identified through the use of PubMed/MEDLINE, Scopus, Web of Science (Science and Social Science Citation Index), and Cochrane Library (Cochrane Database of Systematic Reviews, Cochrane Central Register of Controlled Trials (CENTRAL)) databases.

The combination of non-MeSH/MeSH terms was as follows:

#### 2.1.1. PubMed/MEDLINE

((obese[Title/abstract]) AND (adrenalectomy[Title/abstract])) OR ((obesity[Title/abstract]) AND (adrenalectomy[Title/abstract])) Filters applied: English.

#### 2.1.2. Scopus

(TITLE-ABS-KEY (obese) AND TITLE-ABS-KEY (adrenalectomy) OR TITLE-ABS-KEY (obesity) AND TITLE-ABS-KEY (adrenalectomy)) AND (LIMIT-TO (LANGUAGE, “English”)).

#### 2.1.3. Web of Science

Obese (Topic) AND adrenalectomy (Topic) OR obesity (Topic) AND adrenalectomy (Topic) and English (Languages).

#### 2.1.4. Cochrane Library

Obese in Title Abstract Keyword AND adrenalectomy in Title Abstract Keyword OR obesity in Title Abstract Keyword AND adrenalectomy in Title Abstract Keyword—(Word variations have been searched) Language: English.

Although the first systematic review involved a larger number of terms, just a few results were obtained, and this developed a high risk of ruling out papers of possible interest. Therefore, we decided to restrict our search to the combination obese/obesity + adrenalectomy, in order both to increase the number of results and reduce the risk of excluding manuscripts of interest.

The final analysis was carried out in July 2024. The reference list of the included studies and previously published reviews was screened.

### 2.2. Inclusion Criteria

Our analysis covered case–control studies, case series, cohort studies, randomized clinical trials, and controlled clinical trials, all dealing with adult NOb and Ob patients undergoing RLA for benign or malignant adrenal diseases.

Our study ruled out papers comparing patients undergoing retroperitoneal robotic/open adrenalectomy + RLA or TLA + RLA, in addition to papers comprising fewer than 3 outcomes of interest (see Section 2.3).

We ruled out posters, abstracts, letters to the Editor, editorials, case reports, and previously published systematic reviews and/or meta-analyses. Nevertheless, previously published systematic reviews and/or meta-analyses were included in order to identify comparative studies left out through our systematic search.

As we did not find a high volume of data during our first unsystematic search, in our systematic search, we ruled out restrictions related to the issue date.

### 2.3. Outcomes

Intraoperative and postoperative outcomes were assessed.

The former included the operative time, estimated blood loss (EBL), the transfusion rate, and conversion to open surgery rate.

The latter included the overall postoperative complication rate, the major (Clavien–Dindo or CD ≥ III) postoperative complication rate, and the length of hospital stay.

### 2.4. Data Extraction

The selection and identification of papers were carried out by two independent reviewers (M.Zi. and A.M.), according to titles, abstracts, keywords, and full texts.

Collected data included the following:

- Demographic data [author’s surname and year of publication, study type, study country, study period, population size, gender and age, body mass index (BMI), the American Society of Anesthesiologists (ASA) score, adrenal side, adrenal size, adrenal disease, follow-up duration];
- Intraoperative outcome data [operative time, EBL, transfusion rate, conversion to open surgery rate];
- Postoperative outcome data [overall postoperative complication rate, major (CD ≥ III) postoperative complication rate, length of hospital stay].

In the end, a third independent reviewer analyzed all the collected data (C.G.).

### 2.5. Quality Assessment

The tools used in the quality assessment by the two independent reviewers were the Cochrane Risk-of-Bias tool for randomized trials (Version 2) (RoB 2) and ROBINS-I [24,25].

RoB 2 is recommended in assessing the risk of bias in randomized trials [24]. It includes a fixed set of biases focusing on different aspects of study design, conduct, and reporting [24]. Each domain includes “reporting questions” in order to gather data on study characteristics that could lead to the risk of bias [24]. An algorithm was used to provide a suggestion for bias risk in each domain, according to responses: “low”, “high” or “some concerns” were used as assessment ratings [24].

The ROBINS-I tool is used to assess the risk of bias in non-randomized studies comparing health outcomes in 2 or more interventions [25]. The reporting questions have a factual nature, and their goal is to facilitate judgment on the risk of bias [25]. Answers to those questions serve as a framework for domain-level judgments, which serve as the basis for the overall judgment on the risk of bias in particular outcomes [25]. “Low”, “moderate”, “severe” and “critical” ratings were used, where “low” identified the risk of bias in high-quality randomized studies [25]. The risk of bias in non-randomized studies was rated as “low” in very few cases, because of confounding variables [25].

### 2.6. Statistical Analysis

Our meta-analysis used Review Manager (RevMan) version 5.4 “The Cochrane Collaboration, 2020” [Computer program] [26]. In the case of dichotomous results, 95% confidence intervals (CIs) and odds ratios (ORs) were calculated following the Mantel–Haenszel (MH) method [26]. In the case of continuous outcomes, 95% CIs and weighted mean differences (WMDs) were measured in compliance with the inverse variance (IV) method [26], while in cases without the mean and standard deviation (SD) for an end-point, they were derived from the reported median range and interquartile range (IQR), if available, thanks to Hozo formulas [27]. When sample sizes, means, and SDs were separated for 2 or more subgroups, Cochrane’s formula helped to combine one single sample size, mean, and SD for each group of intervention [28].

I^2^ statistics were used to assess statistical heterogeneity. For this, <25, 25–50, and >50% I^2^ values were classified as follows: low, moderate, and high. Due to the heterogeneity of discrepancies in the general population characteristics, in addition to discrepancies in minimally invasive surgical approaches, a random-effect model was used as default in all statistical analyses. Statistical significance was set at *p* < 0.05.

Publication bias was assessed quantitatively using Egger’s test and visually using funnel plots.

## 3. Results

### 3.1. Search Results

**Figure 1:**
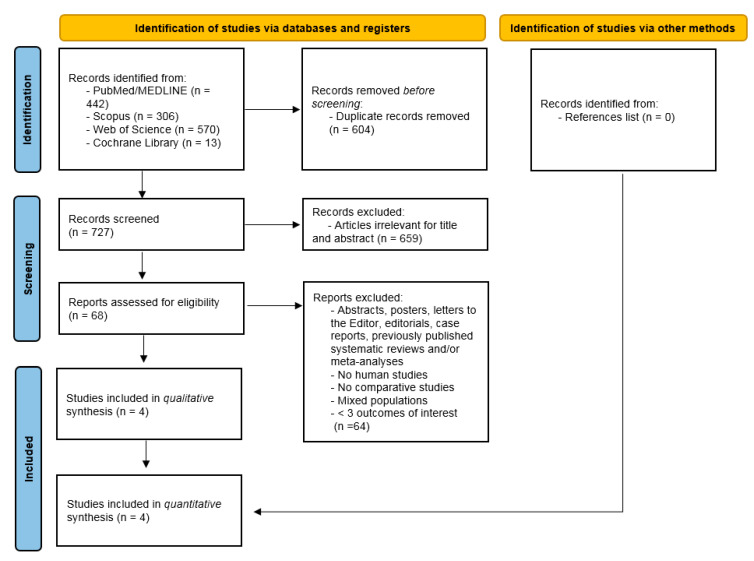
PRISMA flowchart of literature search.

Our final literature search (3 July 2024) led to the identification of 1331 studies (Figure 1).

The removal of both duplicate publications and irrelevant papers for title and abstracts led to the eligibility of 68 full texts, out of which 4 comparative studies complied with inclusion criteria, thus undergoing qualitative and quantitative syntheses [29,30,31,32]. No additional records were found through other sources (list of references).

Incomplete data were integrated through a specific email request to the corresponding authors of the included articles in order to complete the meta-analyses of interest.

### 3.2. Quality of Studies

According to ROBINS-I, all non-randomized studies showed moderate overall bias [29,30,31,32] (see Supplementary Materials, Table S1). The RoB2 tool was not employed, due to the lack of randomized trials.

### 3.3. Study and Population Characteristics

Table 1 shows the study and population features. The four identified studies had an all-observational nature, with a retrospective design. They stemmed from Western and Eastern countries and had an almost 15-year observational time frame (from 2007 to 2022).

The pooled population involved 809 patients, and the sample size ranged between 75 and 353. Sexes were almost equal (48.1% female; 51.9% male); age ranged between 46.11 and 56.7 years, while BMI ranged between 26.3 and 34.1.

Overall, 552 patients were NOb (68.2%), and the sample size ranged between 44 and 290. Sexes were almost equal; age ranged between 48.88 and 56.7 years, while BMI ranged between 24.23 and 26.6.

Additionally, 257 patients were Ob (31.8%), and the sample size ranged between 21 and 122. Sexes were almost equal; age ranged between 46.11 and 55.2 years, while BMI ranged between 32.04 and 36.6.

Table 2 records the surgical and histopathological features of adrenal conditions. Lesions affected the left adrenal gland more than the right one, with the mean diameter ranged between 1.81 and 5.4 cm.

### 3.4. Meta-Analysis Results

#### 3.4.1. Operative Time

**Figure 2:**
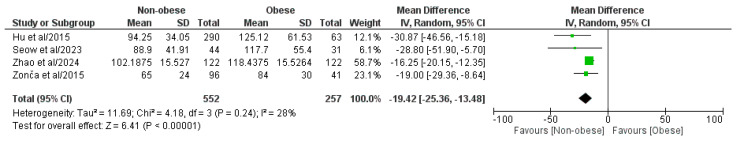
The forest plot comparing the operative time between the NOb and Ob groups. SD, standard deviation; IV, inverse variance; CI, confidence interval [29,30,31,32].

The four included papers, which involved a pooled population of 809 patients, comprising 552 NOb and 257 Ob patients, analyzed the operative time (Figure 2) [29,30,31,32]. The meta-analysis of the pooled results showed that the NOb group had a statistically significantly shorter operative time (MD: −19.42, 95% CI: −25.36, −13.48, *p* < 0.00001), compared to the Ob group. We found a moderate, though statistically negligible, level of heterogeneity (I^2^ = 28%, *p* = 0.24).

#### 3.4.2. Estimated Blood Loss

**Figure 3:**
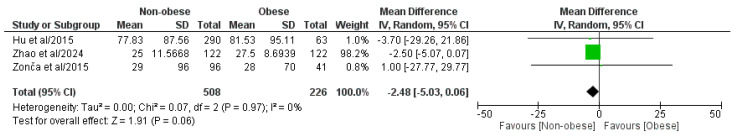
Forest plot comparing estimated blood loss between the NOb and Ob groups. SD, standard deviation; IV, inverse variance; CI, confidence interval [29,30,32].

Three out of the four included studies, which involved a pooled population of 734 patients, comprising 508 NOb and 226 Ob patients, analyzed EBL (Figure 3) [29,30,32]. According to the meta-analysis of the pooled results, statistically significant differences in EBL were not observed between the two groups (MD: −2.48, 95% CI: −5.03, 0.06, *p* = 0.06). We found a low but statistically negligible heterogeneity (I^2^ = 0%, *p* = 0.97).

#### 3.4.3. Transfusion

**Figure 4:**
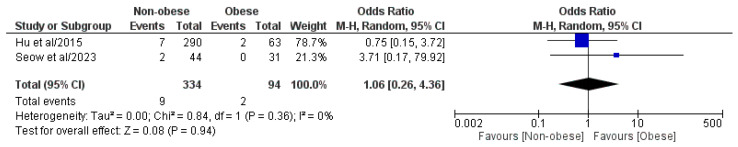
Forest plot comparing reported transfusion rate between the NOb and Ob groups. CI, confidence interval; M-H, Mantel–Haenszel [29,31].

Two out of the four included studies, which involved a pooled population of 428 patients, comprising 334 NOb and 94 Ob patients, analyzed the transfusion rate (Figure 4) [29,31]. According to the meta-analysis of the pooled results, statistically significant differences in the transfusion rate were not observed between the two groups (OR: 1.06, 95% CI: 0.26, 4.36, *p* = 0.94). Heterogeneity had a low level, even if it was statistically negligible (I^2^ = 0%, *p* = 0.36).

#### 3.4.4. Conversion to Open Surgery

**Figure 5:**
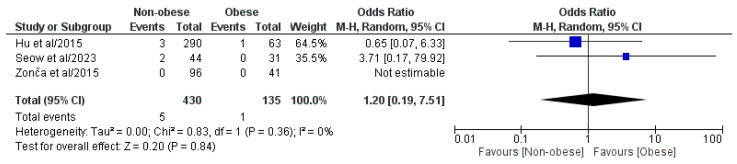
Forest plot comparing reported conversion to open surgery rate between the NOb and Ob groups. CI, confidence interval; M-H, Mantel–Haenszel [29,30,31].

Three out of the four included studies, which involved a pooled population of 565 patients, comprising 430 NOb and 135 Ob patients, analyzed the rate of conversion to open surgery (Figure 5) [29,30,31]. According to the meta-analysis of the pooled results, statistically significant differences in the rate of conversion to open surgery were not observed between the two groups (OR: 1.20, 95% CI: 0.19, 7.51, *p* = 0.84). We found a low, though statistically negligible, level of heterogeneity (I^2^ = 0%, *p* = 0.36).

#### 3.4.5. Overall Postoperative Complications

**Figure 6:**
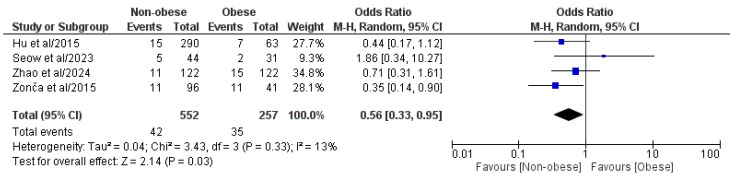
Forest plot comparing the reported overall postoperative complication rate between the NOb and Ob groups. CI, confidence interval; M-H, Mantel–Haenszel [29,30,31,32].

The four included papers involving a pooled population of 809 patients, comprising 552 NOb and 257 Ob patients, analyzed the overall postoperative complication rate (Figure 6) [29,30,31,32]. The meta-analysis of the pooled results showed that the NOb group had a statistically significant lower overall postoperative complication rate (OR: 0.56, 95% CI: 0.33, 0.95, *p* = 0.03), compared to the Ob group. We found a low but statistically negligible heterogeneity (I^2^ = 13%, *p* = 0.33).

#### 3.4.6. Major (Clavien–Dindo or CD ≥ III) Postoperative Complications

**Figure 7:**
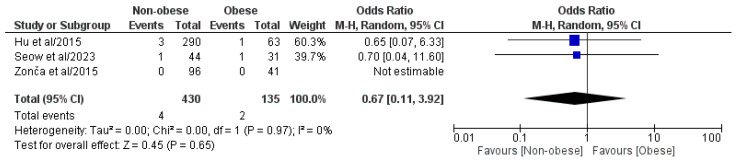
Forest plot comparing the reported major (CD ≥ III) postoperative complication rate between the NOb and Ob groups. CI, confidence interval; M-H, Mantel–Haenszel [29,30,31].

Three out of the four included studies, which involved a pooled population of 565 patients, comprising 430 NOb and 135 Ob patients, analyzed the major (CD ≥ III) postoperative complication rate (Figure 7) [29,30,31]. According to the meta-analysis of the pooled results, statistically significant differences in the major (CD ≥ III) postoperative complication rate were not observed between the two groups (OR: 0.67, 95% CI: 0.11, 3.92, *p* = 0.65). Heterogeneity had a low level, even if it was statistically negligible (I^2^ = 0%, *p* = 0.97).

#### 3.4.7. Length of Hospital Stay

**Figure 8:**
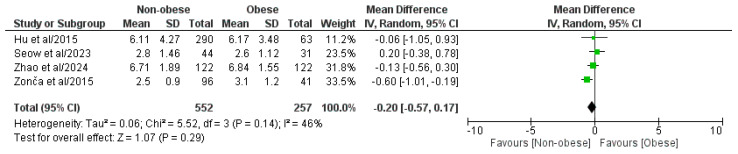
Forest plot comparing the length of hospital stay between the NOb and Ob groups. SD, standard deviation; IV, inverse variance; CI, confidence interval [29,30,31,32].

The four included papers involving a pooled population of 809 patients, comprising 552 NOb and 257 Ob patients, analyzed the length of hospitalization (Figure 8) [29,30,31,32]. According to the meta-analysis of the pooled results, statistically significant differences in the length of hospitalization were not observed between the two groups (MD: −0.20, 95% CI: −0.57, 0.17, *p* = 0.29). We found a moderate, though statistically negligible, level of heterogeneity (I^2^ = 46%, *p* = 0.14).

#### 3.4.8. Subgroup Analysis

Due to discrepancies in study designs, we decided to perform a subgroup analysis. Different outcomes were analyzed, just considering comparative studies with the ≥30 kg/m^2^ obesity criterion. According to subgroup analysis, six out of the seven outcomes of the pooled analysis (see Supplementary Materials, Figures S1–S7) were confirmed. Statistically significant differences were observed in the operative time, which was lower in the NOb group (MD: −23.37, 95% CI: −31.46, −15.27, *p* < 0.00001) (I^2^ = 0%, *p* = 0.41).

#### 3.4.9. Publication Bias

We chose to omit the analysis of publication bias because our meta-analysis involved just four studies. In compliance with the Cochrane Handbook for Systematic Reviews of Interventions (Version 5.1.0), tests for funnel plot asymmetry should be performed in meta-analyses covering at least 10 studies, as a small number of comparative studies reduces the power of tests to identify the case from real asymmetry [33].

## 4. Discussion

Short-term outcomes in NOb and Ob patients undergoing RLA as a consequence of benign or malignant adrenal conditions were assessed in our meta-analysis of comparative studies. All in all, just two meta-analyses investigated the impact of obesity in patients undergoing RLA [20,34]. Danwang et al. analyzed five observational studies: four were related to TLA and just one to RLA [20]. The authors analyzed three parameters (postoperative complications, conversion to open surgery, and readmission within 30 days following surgery) that related to just TLA [20]. No distinction was made between the two approaches, although it became necessary, because of the significant anatomical and surgical discrepancies found in recent years [20]. Xia et al.’s meta-analysis investigated eight observational studies, out of which two concerned RLA [34]. Compared to Danwang et al.’s study, Xia et al.’s meta-analysis involved subgroup analysis concerning RLA (two out of the eight included studies) just in three outcomes (operative time, estimated blood loss, and length of hospital stay) [34]. The operative time and the length of hospital stay turned out to be significantly shorter in the NOb group [34].

Our study on the pooled population of 809 patients (552 patients were NOb and 257 were Ob) who underwent RLA for benign or malignant adrenal diseases revealed statistically significant shorter operative time and lower overall postoperative complications in the NOb group in comparison to the Ob group. Nevertheless, no statistically remarkable discrepancy between the two groups was found, as concerned EBL, transfusion, conversion to open surgery, major (CD ≥ III) postoperative complications, and the length of hospitalization. Taking into account studies with the ≥30 kg/m^2^ obesity criterion, our subgroup analysis confirmed six out of the seven outcomes of the pooled analysis: just the operative time was statistically significantly lower in the NOb group. Therefore, our study showed a lack of significant impact of obesity on intraoperative and postoperative RLA-related morbidity outcomes.

The absence of statistically significant differences in the overall and major postoperative complications in the two groups was a very important result. During individual analysis of the four included comparative studies, only the study by Seow et al. recorded a lower overall complication rate in the Ob group compared to the NOb group, although in the absence of significance [31]. By contrast, as far as the other three studies are concerned, only the one by Zonča et al. showed a significantly higher overall complication rate in the Ob group compared to the NOb group [30]. Furthermore, the overall complication rate in the Ob group in that study was clearly higher than it was in the other three studies [30]. Although such a result can be explained by many factors associated with the heterogeneity of populations under analysis, the main reason was the presence of a high number of secondary tumors in Zonča et al.’s case study (27% NOb and 39% Ob), compared to the other studies that recorded the main presence of benign primary adrenal cases (see Supplementary Materials, Table S2) [29,30,31,32].

Given its fast increase in the global prevalence of overweight, obesity has been considered a risk factor in perioperative complications [35]. Different studies showed that obesity or increased BMI may predict RLA perioperative outcomes, while some studies reported conflicting results [19,29,30,31,32,36]. Our subgroup results showed that the operative time may be significantly related to overweight patients undergoing RLA, according to the BMI criteria (≥30 kg/m^2^).

The possible variability of BMI impact on patient outcomes is worth being underlined. In particular, we may suppose that patients affected by severe obesity (Class III ≥ 40 kg/m^2^) are more likely at risk of intraoperative and postoperative complications, compared to Obesity Class I patients (between 30 and 35 kg/m^2^). Such interesting subgroup analysis could not be carried out, as no included study provided data related to individual obesity classes.

BMI is a reference parameter that shows the obesity level. It does not provide an anthropometric assessment, which may impact the complexity of the retroperitoneal approach, as is the case with the volume of periadrenal or perinephric fat [37].

In the retroperitoneal laparoscopic approach, exposure of the adrenal gland involves three surrounding avascular planes, including the dissection of periadrenal fat and the mobilization of the upper kidney pole down to the hilum [35]. Due to limited space, periadrenal fat has a significant impact on difficult exposure [35].

The presence of adherent perinephric fat can extend the operative time, as it is hardly separated from the renal capsule, thus complicating renal procedures, such as partial nephrectomy [35,38]. Despite the complexity of underlying pathogenesis, inflammation and cardiovascular risk factors may explain the presence of adherent perinephric fat [35,39]. As adipose tissue is an endocrine and immune organ, its impact in producing chronic systemic inflammation has been emphasized for obesity related to insulin resistance and lipid dysregulation [35,40,41]. In metabolic syndrome, above all, an activated cascade of chemokines may cause the infiltration of macrophages into visceral fat, thus mediating fibrosis development and the adhesion of perinephric fat [35,42].

In 2019, Agcaoglu et al. analyzed 82 patients undergoing laparoscopic adrenalectomy [43]. Out of them, 30 patients underwent operation using the retroperitoneal approach [43]. According to multivariate analysis, the authors found that the operative time was linked to BMI in TLA, while the duration of the surgical procedure was related to the thickness of the perinephric fat volume and the distance between the adrenal tumor and the kidney upper pole in RLA [43].

In 2019, Lindeman et al. introduced the Posterior Adiposity Index (PAI), which represents the combination of S-GF (the skin and Gerota’s fascia as distance from the 12th rib tip to Gerota’s fascia) and PNF (the distance between kidney parenchyma and Gerota’s fascia or backside abdominal wall at 12th rib tip) [44]. The PAI has been established as a quantity selection tool in adrenalectomy [44]. By analyzing 56 RLA patients, in a pooled population of 122 patients undergoing minimally invasive adrenalectomy, the authors demonstrated that the PAI was strictly related to the RLA operative time [44].

In 2020, Pearlstein et al. assumed that anthropometric assessments may be better predictors of operative time, in comparison to BMI [37]. In a retrospective analysis of an 83-patient population undergoing RLA for different adrenal lesions, the authors found that periadrenal fat volume (at abdomen CT scan) was significantly associated with the operative time using multivariable analysis [37]. The PAI turned out as a significant predictor of the operative time in univariable analysis, although it did not maintain its significance in multivariable analysis, when tumor laterality, lesion histology, surgeon experience, periadrenal fat volume, and other variables were taken into account [37].

The Mayo adhesive probability score (MAP or Mayo score) represents an image-based scoring system first used to preoperatively assess the presence of troublesome adherent fat during robot-assisted partial nephrectomy [45]. This ready-to-use risk scoring system includes only two radiological factors, i.e., background perinephric fat thickness and perinephric fat stranding type [45]. The perinephric fat profile, represented by the Mayo score, is involved in RLA [45].

In 2022, Chen et al. retrospectively reviewed patients who underwent RLA in the period between 2017 and 2020 for the diagnosis of benign adrenal tumors at their institution [35]. In that study, 186 RLA patients were included [35]. The authors found that a longer operative time, higher estimated blood loss, and higher levels of hemoglobin decline were significantly associated with higher Mayo score risks [35]. No different complication rates were found [35]. The MAP score was considered as the sole, independent risk factor for perioperative outcomes on multivariable analysis [35].

Similar conclusions were reached by Yuan et al. in their retrospective analysis of 104 patients undergoing RLA [46]. The authors discovered that patients marked by high MAP scores showed statistically significantly longer operative time, drainage tube removal time, and higher EBL [46]. Moreover, high MAP scores and tumor size were independent risk factors for longer operative time, while a higher BMI and larger tumor size were independent risk factors in high MAP scores [46]. Therefore, the authors came to the conclusion that the MAP score was related to RLA perioperative outcomes, while BMI and tumor size were better indicators of the MAP score, as they affected RLA complexity [46].

Many factors may have affected the aforementioned meta-analysis results more or less significantly, all of which we have previously discussed [47]: (i) the laterality and size of the adrenal lesion; (ii) surgically treated adrenal conditions; and (iii) the surgeon learning curve. However, none of the studies included in our meta-analysis investigated the individual impact of the aforementioned factors, making conclusions on this matter hard to draw.

An interesting peculiarity that requires further investigation concerned patients with cortisol-secreting tumors and affected by Cushing’s syndrome: they must be considered different from other patients affected by a benign primary adrenal disease. Their presence in the study population could have affected the final results.

According to the scientific literature, Cushing’s syndrome represents a serious endocrine condition related to increased mortality risk and threat to life quality, due to the onset of more or less severe comorbidities [48,49,50]. They include metabolic syndromes (hypertension, visceral obesity, impaired glucose metabolism, and dyslipidemia); musculoskeletal disorders (myopathy, osteoporosis, and skeletal fractures); neuropsychiatric conditions; and impaired reproductive and sexual functions and dermatological manifestations [48,49,50].

Hypertension in patients with Cushing’s syndrome contributes to an increased risk of myocardial infarction, heart failure, or stroke, which are the most common causes of death and whose risks are increased by prothrombotic diathesis and hypokalemia [48,49,50]. Immune disorders are also common, as immunosuppression during active disease gives rise to infection susceptibility, possibly complicated by sepsis, which is a major cause of death [48,49,50].

Two consequences related to the syndrome should be taken into account: (i) visceral obesity, which is peculiar to the pathology and strongly impacts the BMI of the patient affected by Cushing’s syndrome, might have been the determining factor in the number of severely obese patients (Class II 35.0–39.9 and Class III ≥ 40.0) in the individual populations under examination; (ii) comorbidities that are peculiar to patients with Cushing’s syndrome could have influenced the overall rate of postoperative complications in the individual populations under examination. Given the paucity of patients with Cushing’s syndrome in the populations, none of the four included studies analyzed this topic, thus making it impossible to draw any conclusion.

Missing adrenocortical carcinomas treated with RLA in the pooled population must be kept in mind. Current scientific papers underline how RLA and TLA yield similar outcomes, as regards the histology of the treated adrenal lesions [51,52,53,54,55,56]. Non-functioning and functioning benign lesions and secondary tumors are effectively treated using both methods [51,52,53,54,55,56]. However, in the presence of malignant primary adrenal lesions such as adrenocortical carcinoma, the rupture of the lesion must be ruled out, and/or simultaneous lymphadenectomy (in selected cases) with respect to oncological radicality must be carried out [4,5,6,47,57]. In clear or alleged primary malignancies, only a few authors suggest using both methods in selected cases and in high-volume centers, while many scientific societies strongly recommend an open approach [4,5,6,57,58,59,60,61].

The present meta-analysis has several non-negligible limitations. No randomized controlled study was identified, with the exception of observational studies, just one of which had a propensity-score matching analysis. The number of included studies and enrolled patients was small. Moreover, the general population and surgically treated adrenal disease features (as concerned histology, side, and size) were heterogeneous.

To the best of our knowledge, our study reached the best evidence on this topic, as we included just comparative (double-arm) studies on RLA in both NOb and Ob populations. Danwang et al.’s meta-analysis included five studies, four of which were related to TLA, and just one involved RLA (353 patients) [20]. Similarly, Xia et al.’s meta-analysis included eight studies, out of which two studies involved RLA (490 patients) [34]. Furthermore, limited to just the two RLA studies, Xia et al. provided a subgroup analysis of three outcomes [34]. Our meta-analysis concentrated exclusively on populations who underwent RLA in a total of four comparative studies, including an almost double-pooled population (809 patients) compared to Xia et al.’s study, and a significantly higher number of outcomes were analyzed in our analysis (seven) [34].

## 5. Conclusions

By including comparative studies of NOb and Ob adult patients undergoing RLA for benign or malignant adrenal disorders, our meta-analysis showed that RLA involved statistically different operative time and overall postoperative complications in the two populations, in favor of the NOb group. Considering the subgroup analysis (≥30 kg/m^2^), just the operative time maintained statistical significance, in the absence of discrepancies regarding the intraoperative and postoperative morbidity results. To the best of our knowledge, obesity did not appear to impact RLA safety and effectiveness.

Our results need cautious assessment due to significant biases among the studies included in the meta-analysis, the small overall sample size, and the paucity of the analyzed events. Further well-designed randomized multicenter controlled studies are necessary to confirm our results and obtain a proper selection of patients.

In particular, it would be useful to carry out studies on the impact of obesity by referring not only to BMI but to different anthropometric measures already known in the literature.

## Figures and Tables

**Table 1:** Study and population characteristics.

Authors/Year	Study Type	Study Country	Study Period	Group	Patient Population, n	Gender, n		Age (Years), Mean ± SD	BMI (kg/m^2^), Mean ± SD	**Obesity Criteria**	**ASA Score, n**		**FU** **(Months), Mean ± SD**
						Male	Female				**I–II**	**III–IV**	
Hu et al./2015 [29]	Retrospective	China	2011–2013	Obese	63	34	29	46.11 ± 11.83	32.04 ± 2.97	≥30 kg/m^2^	44	19	until 30 days after surgery
				Non-obese	290	136	154	48.88 ± 12.35	24.23 ± 2.77		251	39	
Zonča et al./2015 [30]	Retrospective	Germany, Czech Republic	2007–2013	Obese	41	16	25	53 ± 14	34.1 ± 3.5	≥30 kg/m^2^	26	15	n/a
				Non-obese	96	47	49	51 ± 15	26.6 ± 2.1		84	12	
Seow et al./2023 [31]	Retrospective	Australia	2014–2022	Obese	31	11	20	55.2 ± 12.59	36.6 ± 6.88	≥30 kg/m^2^	14	17	50.4 ± 26.8
				Non-obese	44	28	16	56.7 ± 13.04	26.3 ± 2.96		24	20	
Zhao et al./2024 [32]	Retrospective PSM	China	2012–2022	Obese	122	72	50	51.52 ± 11.50	n/a	≥28 kg/m^2^	83	39	n/a
				Non-obese	122	76	46	49.65 ± 12.61	n/a		82	40	

n = number; SD = standard deviation; BMI = body mass index; ASA = American Society of Anesthesiologists; FU = follow-up; n/a = not available; PSM = propensity score-matched analysis.

**Table 2:** Surgical and histopathological characteristics of adrenal pathology.

Authors/Year	Group	Adrenal Side, n			Adrenal Lesion Size (cm), Mean ± SD	Adrenal Disease, n					
						Benign Lesions					Malignant Lesions
		Right	Left	Bilateral		Aldosterone-Secreting Tumors	Catecholamine-Secreting Tumors	Glucocorticosteroid-Secreting Tumors	Hormonally Inactive/Non-Functioning Tumors	Other ^	Secondary Tumors
Hu et al./2015 [29]	Obese	30	33	0	2.67 ± 1.49	22	20	15	6	0	0
	Non-obese	134	156	0	2.74 ± 1.30	97	108	50	35	0	0
Zonča et al./2015 [30]	Obese	n/a	n/a	n/a	4.7 ± 1.6	6	10	3	5	1	16
	Non-obese	n/a	n/a	n/a	5.4 ± 2.3	20	30	9	8	3	26
Seow et al./2023 [31]	Obese	37	34	3	5.13 ± 2.87	18	10	4	34	0	9
	Non-obese			1	5.08 ± 2.58						
Zhao et al./2024 [32]	Obese	43	79	0	1.85 ± 0.3082	122 *					0
	Non-obese	44	78	0	1.8125 ± 0.3897	122 *					0

n = number; SD = standard deviation; n/a = not available. ^ = virilizing adenoma (2 NOb vs. 0 Ob), angiomyolipoma (1 NOb vs. 1 Ob). * = 86 adenoma cases + 36 hyperplasia cases in each group.

## Data Availability

The data presented in this study are available upon request from the corresponding author.

## Supplementary Materials

The following supporting information can be downloaded at: https://www.mdpi.com/article/10.3390/medicina61010106/s1, Table S1. Retrospective studies evaluated using ROBINS-I.; Table S2. Overall postoperative complications.; Figure S1. Forest plot comparing the operative time between the NOb and Ob groups [obesity ≥ 30 kg/m^2^ subgroups]. SD, standard deviation; IV, inverse variance; CI, confidence interval.; Figure S2. Forest plot comparing estimated blood loss between the NOb and Ob groups [obesity ≥ 30 kg/m^2^ subgroups]. SD, standard deviation; IV, inverse variance; CI, confidence interval.; Figure S3. Forest plot comparing reported transfusion rate between the NOb and Ob groups [obesity ≥ 30 kg/m^2^ subgroups]. CI, confidence interval; M-H, Mantel–Haenszel.; Figure S4. Forest plot comparing reported conversion to open surgery rate between the NOb and Ob groups [obesity ≥ 30 kg/m^2^ subgroups]. CI, confidence interval; M-H, Mantel–Haenszel.; Figure S5. Forest plot comparing reported the overall postoperative complication rate between the NOb and Ob groups [obesity ≥ 30 kg/m^2^ subgroups]. CI, confidence interval; M-H, Mantel–Haenszel.; Figure S6. Forest plot comparing reported the major (CD ≥ III) postoperative complication rate between the NOb and Ob groups [obesity ≥ 30 kg/m^2^ subgroups]. CI, confidence interval; M-H, Mantel–Haenszel.; Figure S7. Forest plot comparing the length of hospital stay between the NOb and Ob groups [obesity ≥ 30 kg/m^2^ subgroups]. SD, standard deviation; IV, inverse variance; CI, confidence interval.
