# Supplementary material for: Impact of Obesity on Short-Term Outcomes in Patients Undergoing Retroperitoneal Laparoscopic/Retroperitoneoscopic Adrenalectomy for Benign or Malignant Adrenal Diseases: A Meta-Analysis
Source: Medicina (Kaunas). 2025 Jan 13;61(1):106. doi: 10.3390/medicina61010106 (PMC11766650; doi:10.3390/medicina61010106)
Supplement: Supplementary file 1 [file medicina-61-00106-s001.zip › medicina-3244107-supplementary.pdf]

Supplementary material

Tables S1. Retrospective studies evaluated using ROBINS-I.

		**Hu et al/2015 [29]**	**Zonça et al/2015 [30]**	**Seow et al/2023 [31]**	**Zhao et al/2024 [32]**
Preintervention	Confounding	Moderate	Moderate	Moderate	Moderate
	Selection bias	Low	No information	Low	No information
Intraintervention	Classification of interventions	Low	Low	Low	Low
Postintervention	Intended interventions	Low	Low	Low	Low
	Missing data	Low	Low	Low	Low
	Measurement of outcomes	Low	Low	Low	Low
	Reported results	Low	Low	Low	Low
Overall bias		Moderate	Moderate	Moderate	Moderate

Tables S2. Overall postoperative complications.

<table>
<tr><th>Authors/Year</th><th>Group</th><th>Overall complications, n (%)</th><th>p value</th><th>Superficial SSI, n</th><th>Deep SSI, n</th><th>Trasfusions, n</th><th>Pneumonia, n</th><th>Atelectasis, n</th><th>Urinary retention, n</th><th>Incisional hernia, n</th><th>Other, n</th></tr>
<tr><td rowspan="2">Hu et al/2015 [29]</td><td>Non-obese</td><td>15 (8.6)</td><td rowspan="2">0.208</td><td>5</td><td>2</td><td>7</td><td>1</td><td></td><td></td><td></td><td></td></tr>
<tr><td>Obese</td><td>7 (12.5)</td><td>4</td><td>1</td><td>2</td><td>0</td><td></td><td></td><td></td><td></td></tr>
<tr><td rowspan="2">Zonça et al/2015 [30]</td><td>Non-obese</td><td>11 (11.5)</td><td rowspan="2">0.025</td><td>4</td><td></td><td></td><td>2</td><td>2</td><td>3</td><td></td><td></td></tr>
<tr><td>Obese</td><td>11 (26.8)</td><td>4</td><td></td><td></td><td>0</td><td>5</td><td>2</td><td></td><td></td></tr>
<tr><td rowspan="2">Seow et al/2023 [31]</td><td>Non-obese</td><td>5 (11.4)</td><td rowspan="2">0.471</td><td colspan="2">1</td><td>2</td><td></td><td></td><td></td><td>1</td><td>1</td></tr>
<tr><td>Obese</td><td>2 (6.5)</td><td colspan="2">0</td><td>0</td><td></td><td></td><td></td><td>1</td><td>1</td></tr>
<tr><td rowspan="2">Zhao et al/2024 [32]</td><td>Non-obese</td><td>11 (9)</td><td rowspan="2">0.41</td><td>n/a</td><td>n/a</td><td>n/a</td><td>n/a</td><td>n/a</td><td>n/a</td><td>n/a</td><td>n/a</td></tr>
<tr><td>Obese</td><td>15 (12.3)</td><td>n/a</td><td>n/a</td><td>n/a</td><td>n/a</td><td>n/a</td><td>n/a</td><td>n/a</td><td>n/a</td></tr>
</table>

Subgroup analysis with ≥ 30 kg/m² obesity criteria studies

	Non-obese			Obese				Mean Difference
**Study or Subgroup**	**Mean**	**SD**	**Total**	**Mean**	**SD**	**Total**	**Weight**	**IV, Random, 95% CI**
Hu et al/2015	94.25	34.05	290	125.12	61.53	63	26.6%	-30.87 [-46.56, -15.18]
Seow et al/2023	88.9	41.91	44	117.7	55.4	31	12.3%	-28.80 [-51.90, -5.70]
Zonča et al/2015	65	24	96	84	30	41	61.1%	-19.00 [-29.36, -8.64]
**Total (95% CI)**			**430**			**135**	**100.0%**	**-23.37 [-31.46, -15.27]**

Heterogeneity: $Tau^2 = 0.00$; $Chi^2 = 1.77$, df = 2 (P = 0.41); $I^2 = 0\%$

Test for overall effect: Z = 5.66 (P < 0.00001)

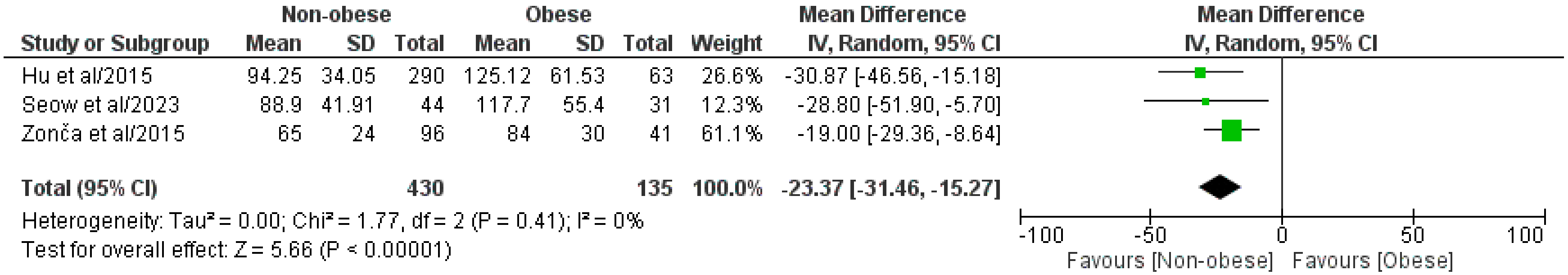

Figure S1. Forest plot comparing **operative time** between the NOb and Ob groups [Obesity ≥ 30 Kg/m² subgroups]. SD, Standard Deviation; IV, inverse variance; CI, confidence interval. [29-31]

	Non-obese			Obese				Mean Difference
**Study or Subgroup**	**Mean**	**SD**	**Total**	**Mean**	**SD**	**Total**	**Weight**	**IV, Random, 95% CI**
Hu et al/2015	77.83	87.56	290	81.53	95.11	63	55.9%	-3.70 [-29.26, 21.86]
Zonča et al/2015	29	96	96	28	70	41	44.1%	1.00 [-27.77, 29.77]
**Total (95% CI)**			**386**			**104**	**100.0%**	**-1.63 [-20.73, 17.48]**

Heterogeneity: $Tau^2 = 0.00$; $Chi^2 = 0.06$, df = 1 (P = 0.81); $I^2 = 0\%$

Test for overall effect: Z = 0.17 (P = 0.87)

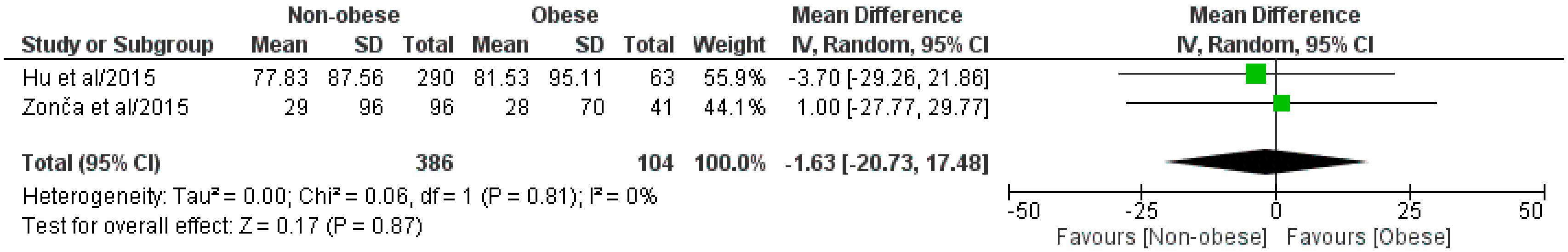

Figure S2. Forest plot comparing **estimated blood loss** between the NOb and Ob groups [Obesity ≥ 30 Kg/m² subgroups]. SD, Standard Deviation; IV, inverse variance; CI, confidence interval. [29,30]

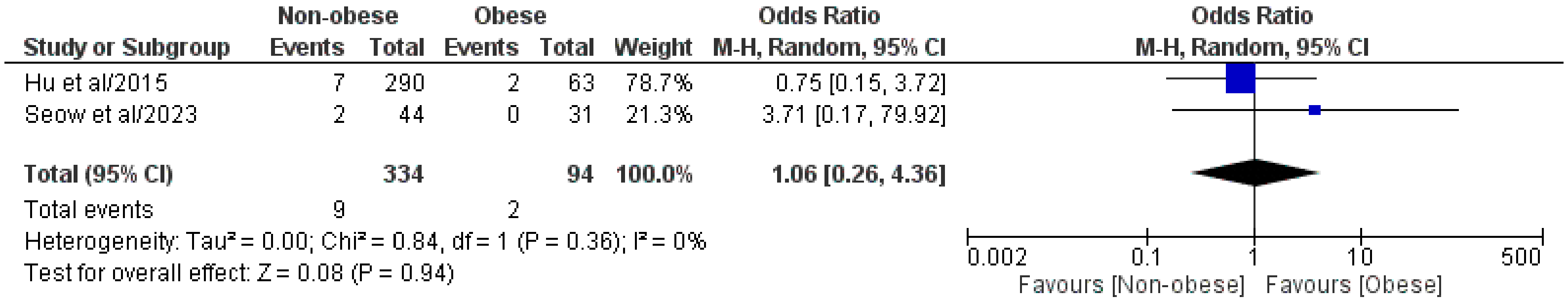

Figure S3. Forest plot comparing reported **transfusion rate** between the NOb and Ob groups [Obesity ≥ 30 Kg/m² subgroups]. CI, confidence interval; M-H, Mantel–Haenszel. [29,31]

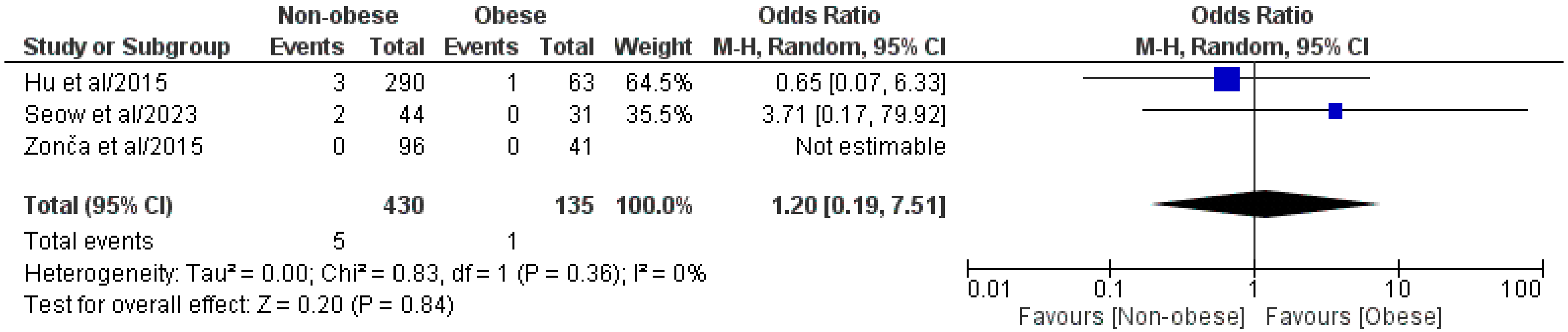

Figure S4. Forest plot comparing reported **conversion to open surgery rate** between the NOb and Ob groups [Obesity ≥ 30 Kg/m² subgroups]. CI, confidence interval; M-H, Mantel–Haenszel. [29-31]

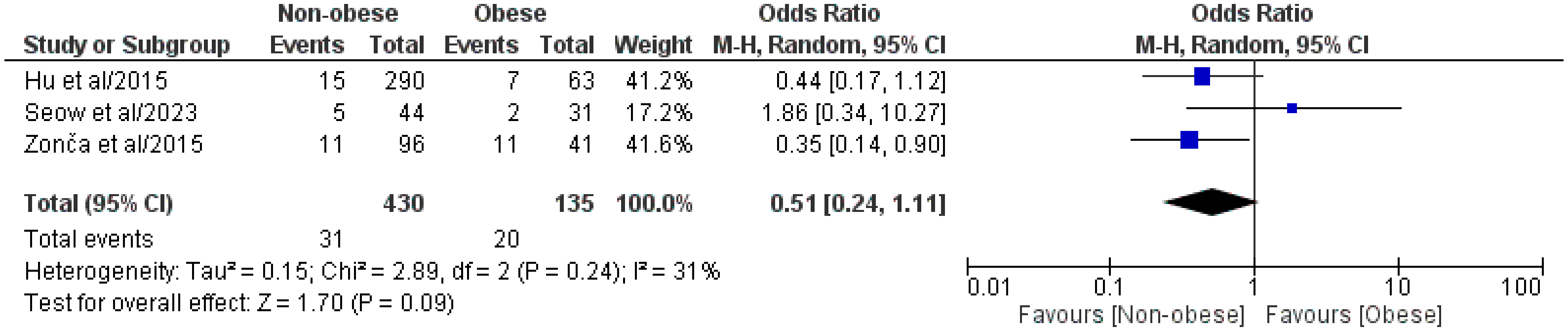

Figure S5. Forest plot comparing reported **overall postoperative complications rate** between the NOb and Ob groups [Obesity ≥ 30 Kg/m² subgroups]. CI, confidence interval; M-H, Mantel–Haenszel. [29-31]

	Non-obese		Obese			Odds Ratio
**Study or Subgroup**	**Events**	**Total**	**Events**	**Total**	**Weight**	**M-H, Random, 95% CI**
Hu et al/2015	3	290	1	63	60.3%	0.65 [0.07, 6.33]
Seow et al/2023	1	44	1	31	39.7%	0.70 [0.04, 11.60]
Zonča et al/2015	0	96	0	41		Not estimable
**Total (95% CI)**		**430**		**135**	**100.0%**	**0.67 [0.11, 3.92]**
Total events	4		2			

Heterogeneity: Tau² = 0.00; Chi² = 0.00, df = 1 (P = 0.97); I² = 0%
Test for overall effect: Z = 0.45 (P = 0.65)

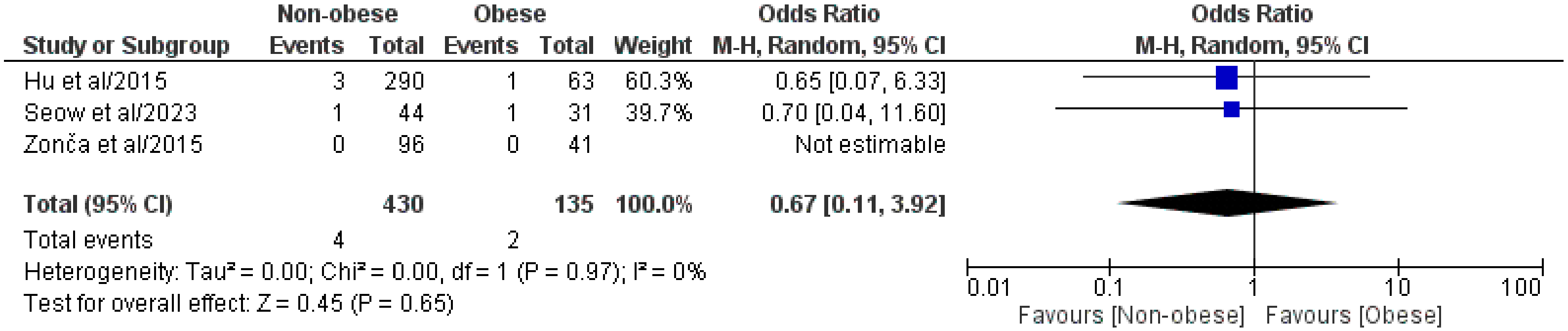

Figure S6. Forest plot comparing reported **major (CD ≥ III) postoperative complications rate** between the NOb and Ob groups [Obesity ≥ 30 Kg/m² subgroups]. CI, confidence interval; M-H, Mantel–Haenszel. [29-31]

	Non-obese			Obese				Mean Difference
**Study or Subgroup**	**Mean**	**SD**	**Total**	**Mean**	**SD**	**Total**	**Weight**	**IV, Random, 95% CI**
Hu et al/2015	6.11	4.27	290	6.17	3.48	63	20.9%	-0.06 [-1.05, 0.93]
Seow et al/2023	2.8	1.46	44	2.6	1.12	31	35.4%	0.20 [-0.38, 0.78]
Zonča et al/2015	2.5	0.9	96	3.1	1.2	41	43.7%	-0.60 [-1.01, -0.19]
**Total (95% CI)**			**430**			**135**	**100.0%**	**-0.20 [-0.77, 0.37]**

Heterogeneity: Tau² = 0.15; Chi² = 5.10, df = 2 (P = 0.08); I² = 61%
Test for overall effect: Z = 0.70 (P = 0.48)

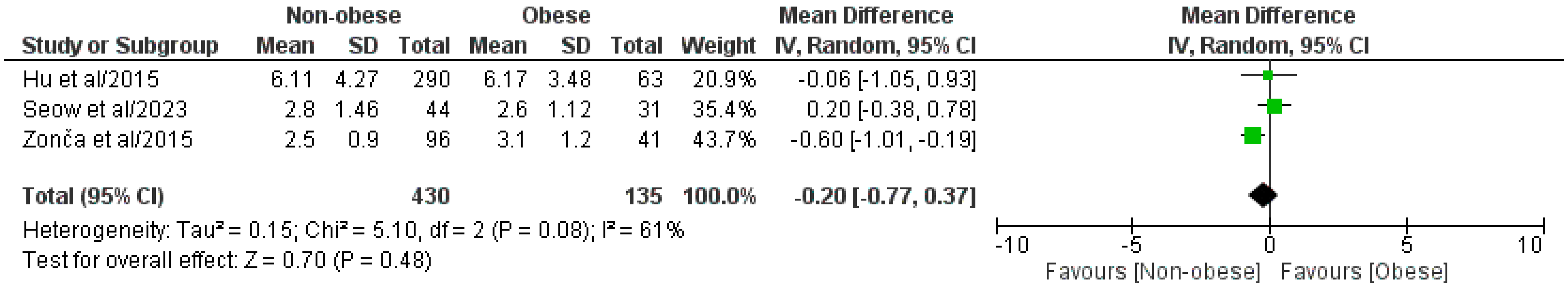

Figure S7. Forest plot comparing **length of hospital stay** between the NOb and Ob groups [Obesity ≥ 30 Kg/m² subgroups]. SD, Standard Deviation; IV, inverse variance; CI, confidence interval. [29-31]
